# Impact of chitosan-incorporated toothpaste on roughness, gloss, and antifungal potential of acrylic resin

**DOI:** 10.1038/s41598-023-47530-w

**Published:** 2023-12-04

**Authors:** Kaye Varaschin Theodorovicz, Waldemir Franscisco Vieira-Junior, Raissa Manoel Garcia, Ludmila Pini Simões Gobbi, Mariana Mayume Mori, Benedito Prado Dias Filho, Débora Alves Nunes Leite Lima, Daniel Sundfeld, Núbia Inocencya Pavesi Pini

**Affiliations:** 1 Department of Odontology, Pato Branco University Center (UNIDEP), Pato Branco, PR Brazil; 2 Department of Restorative Dentistry, São Leopoldo Mandic Institute and Dental Research Center (SLMandic), Campinas, SP Brazil; 3 Department of Restorative Dentistry, Piracicaba Dental School, University of Campinas (FOP/UNICAMP), Piracicaba, SP Brazil; 4 Department of Pharmaceutical Sciences, State University of Maringá (UEM), Maringá, PR Brazil; 5 Department of Restorative and Prosthetic Dentistry, Ingá University Center (UNINGÁ), Estrada PR 317, 6114 – Parque Industrial 200, Maringá, PR 87035-510 Brazil

**Keywords:** Biotechnology, Microbiology

## Abstract

This study aimed to test the efficacy of different silica-based toothpastes with or without chitosan, as a method of cleaning the acrylic surfaces of denture prostheses. Acrylic resin specimens were prepared to evaluate surface roughness and gloss (n = 10), and *Candida albicans* adhesion/inhibition (n = 2). Two toothpastes with different degrees of abrasiveness were used: Colgate (CT) and Elmex (EX), with or without 0.5% chitosan (Ch) microparticles (CTCh or EXCh, respectively). The negative control was brushed with distilled water. Brushing was simulated with a machine. Surface roughness and gloss were analyzed before and after brushing. *Candida albicans* incidence/inhibition was tested qualitatively to determine the acrylic resin antifungal activity. The roughness and gloss data were analyzed with a generalized linear model, and the Kruskal Wallis and Dunn tests, respectively (α = 5%). Brushing with toothpastes increased roughness and reduced gloss, compared with the negative control (*p* < 0.05). CT showed a more significantly different change in roughness and gloss, in relation to the other groups (*p* < 0.05). Addition of chitosan to CT reduced its abrasive potential, and yielded results similar to those of EX and EXCh. Specimens brushed with CT showed a higher potential for *Candida albicans* adherence, despite its higher antifungal action. Addition of chitosan to the toothpaste made both toothpaste and brushing more effective in inhibiting *Candida albicans*. CT had the potential to increase roughness, reduce gloss, and increase *Candida albicans* adherence. In contrast, chitosan added to CT showed greater antifungal potential, and a higher synergistic effect than EX.

## Introduction

Acrylic resin is the material most commonly used as a base for complete or partial removable dentures, and for implant support dentures, such as protocol prostheses and overdentures^1–3^. This resin must remain in close contact with the oral tissues in order to promote the correct mastication function, and be esthetically acceptable to the patient^4^. Since these protheses call for long-term use, their acrylic surface must be maintained properly polished and smooth, considering that retention of biofilm would make the surface irregular^5–7^. Commonly, patients who wear acrylic prosthesis are prone to present with oral stomatitis, resulting from retention of *Candida albicans* fungus in contact with the oral mucosa^6,8–10^. Despite to be a multifatorial condition, which can be modulated by the saliva of the patient considering its bacterial and protein content, pH, among other factors, which, by the way, may be influenced by sytemical conditions and use of specific drugs, clinicians must be able to prevent this condition in these patients (REF). In this respect, dental practitioners must advise patients of the proper hygiene protocols to prevent this situation.

In the case of removable prosthesis, the denture surface must be cleaned with a toothbrush, water and neutral soap^3,11–13^. However, this procedure cannot be used for fixed implant-supported prostheses; instead, the most common oral care procedure is to brush with regular toothpaste^14–16^. On the other hand, using toothpaste raises an important concern related to its abrasiveness. Several studies have shown that toothbrush and toothpaste may damage the surface of the acrylic resin, dull the polish, increase roughness and reduce gloss^15,17–19^. As a consequence, the material can retain more microbiological biofilm^3^. Unfortunately, this occurs because acrylic resins have lower resistance to the abrasive forces promoted by brushing with different bristles, and using toothpastes with different compositions and degrees of abrasiveness^20,21^.

This concern can be addressed by selecting an adequate toothpaste. Currently, different toothpastes are available with a variety of ingredients and degrees of abrasiveness, and some have antimicrobial agents. In this respect, chitosan is a biopolymer that has been tested in the dental field for its antimicrobial effect, remineralizing ability and lubricant properties^22–27^. Its antifungal potential has tested effective in inhibiting the adhesion and development of *Candida albicans* biofilm^22^, and has proved suitable for use. However, to the best of the authors’ knowledge, there is no study that evaluates the potential of chitosan-incorporated toothpaste in reducing the changes in the acrylic surface of denture prostheses, or in inhibiting fungal growth on the protheses.

This study aimed to test the hygiene protocols indicated for patients who wear acrylic based prostheses, by varying the toothpaste used, and assessing the addition versus non-addition of chitosan microparticles. Other objectives included evaluation of surface roughness and gloss using resin surfaces treated with chitosan, and effect of chitosan on the tested products. Besides, the analysis of *Candida albicans* incidence and inhibition potential of *Candida albicans* by the agar well diffusion method were performed. The following null hypotheses was tested: the toothpastes used are not significantly different in regard to effecting changes in the surface roughness or gloss of the acrylic material.

## Material and methods

### Experimental design

The study was conducted based on two experiments: a quantitative one (n = 10/group) to evaluate surface roughness and gloss, and a qualitative one (n = 2/group) to analyze the antifungal potential of the toothpastes tested. Two different silica-based toothpastes with varying degree of abrasiveness, and with or without the addition of chitosan were researched. Chitosan was used in the form of a microparticle obtained from the primary molecule using spray dryer technology^28^. The negative control treatment consisted of brushing with distilled water. The following study groups were established: NC—negative control: brushing with distilled water; CT: brushing with Colgate toothpaste; CTCh: brushing with Colgate toothpaste combined with 0.5% chitosan; EX: brushing with Elmex toothpaste; EXCh: brushing with Elmex toothpaste combined with 0.5% chitosan. The products used are described in Table 1.

**Table 1:** Products used in the study.

Material	Composition	Manufacturer
VIPI CRIL PLUS heat cure acrylic resin	Powder: polymethyl methacrylate, benzoyl peroxide, pigments Liquid: methyl methacrylate, EDMA (crosslink), fluorescent inhibitor	VIPI industry, commerce, export and import of dental products ltd., Pirassununga, São Paulo, Brazil
Colgate Total 12 toothpaste (CT)	Sodium fluoride (1450 ppm fluoride ion), 0.3% Triclosan, sodium lauryl sulfate, sorbitol, hydrated silica, Gantrez, sodium saccharin, flavor, dyes, water, carrageenan, sodium hydroxide, titanium dioxide, artificial dyes CI 77891, CI 77019, and CI 42090 RDA: 100 ± 5^44^	Colgate-Palmolive Industry Ltd., São Bernardo do Campo, São Paulo, Brazil
Elmex anticaries protection toothpaste (EX)	Aqua, Hydrated Silica, Sorbitol, Hydroxyethyl cellulose, Olaflur, Aroma, Saccharin, CI 77891, Limonene RDA: 69 ± 5^44^	Colgate-Palmolive Industry Ltd., São Bernardo do Campo, São Paulo, Brazil
Chitosan (Ch)	Medium molecular weight 300–400 kDA; Degree of deacetylation of 75–85% CAS, 9012-76	Sigma-Aldrich Marginal Pinheiros, 23043, São Paulo, SP, Brazil

### Specimen preparation

Thermopolymerizable acrylic resin (VipiCril Plus; VIPI, Pirassununga, Brazil) was used. Sixty resin discs 10 mm in diameter and 3.0 ± 0.1 mm thick were made using metal molds. The resin was manipulated according to the manufacturer’s instructions, inserted into the mold, and submitted to a high-capacity hydraulic pressure unit (Protecni no. 2; Protecni, São Paulo, Brazil) under 40 lbs., at 100 °C, for 30 min. The resin was polymerized, the excess was removed using a trimming bur, and the specimens were stored in distilled water for 48 h, at 37 °C. Finishing and polishing were performed by a single operator using an electric motor-driven handpiece with a silicon carbide bar (NTI; Kahla, Germany) applied for 15 s. Polishing was performed using specific brushes for acrylic resin in decreasing granulations (high, medium and low—DHPro, Paranaguá, Brazil), followed by a soft brush and polishing paste (Fotoacril; DHPro, Paranaguá, Brazil). After the preparation, the specimens were sterilized under controlled temperature (129 °C) and individually stored in specific recipients until use. Initially, the samples were submitted to a baseline analysis of roughness and gloss, according to which they were allocated randomly among the pre-assigned experimental groups (n = 10).

### Hygiene protocols

After allocation of the specimens, they were brushed with toothpaste slurries prepared with distilled water at a ratio of 1:3 (g/ml), with or without the addition of 0.5% chitosan. The suspensions were always freshly prepared, and homogenized in a magnetic stirrer for 5 min at room temperature, immediately before use. For each specimen, it was used 20 ml of toothpaste slurry, at the initial of the brushing test. During the test, the slurry was renewed if necessary.

The toothbrushes (Oral-B PRO-SAÚDE no. 40; Oral-B, Seropédica, Brazil) were prepared for brushing by cutting them at the neck, and then attaching them to the brush support of the equipment (ODEME MEV 3T—10XY; ODEME, Luzerna, Brazil) with screws placed on the sides and at the top of the brush. The specimens were submitted to 10,000 cycles applying a back-and-forth movement, at a rate of 60 reciprocal strokes per minute, with an amplitude of 25 mm, under 200 g of vertical load on each specimen. The simulated brushing used an in vitro method that has been considered suitable for quantifying acrylic resin abrasiveness according to ISO/TR14569_1:2007. The brushing cycle was monitored, and the toothpaste slurry was renewed every 15 min. Subsequent to brushing, the specimens were washed in distilled water and stored individually.

### Analysis of surface roughness

The roughness analysis was performed twice: at baseline (initial time point) and after brushing (final time point). A surface profile-measuring device (Surfitest 211; Mitutoyo, Kawasaki, Japan) was used. The readings were made in 3 different positions, turning 120° at each measurement to obtain the roughness average (Ra) of each specimen. The analysis was performed with a 0.25-mm cutoff, 5 N static load, 3-mm runup distance, and speed of 0.5 mm/s. The variation in roughness values (**∆**Ra) was calculated between the final and initial measurements to assess possible changes in the surface profile.

### Analysis of surface gloss

The analysis of surface gloss was also performed at baseline (initial) and after brushing (final), using a glossmeter (Novo-Curve; Rhopoint Instruments, St. Leonards-on-Sea, UK), which projects a light beam onto the surface of the specimen at a 60° angle (ISO-Standards, ISSO 2813). The device has a 4.5-mm aperture, and was calibrated (93.3 GU) with a plate provided by the manufacturer, before starting the tests. Four measurements were made, corresponding to each quadrant of the specimen. The mean reading was recorded as a unit of gloss. The variation in this analysis (**∆**GU) was also calculated between the final and initial measurements.

### Antifungal analysis

The inoculum used for the antifungal analysis was *C. albicans* species ATCC 10231, which was re-streaked on a Sabouraud dextrose agar plate (KAVI), and incubated for 24–48 h, under 37 °C. A Sabouraud dextrose broth suspension was made to produce a final density of 10^6^ CFU/mL (colony forming unit/milliliter)^28^.

### Analysis of *C. albicans* incidence

The incidence of *C. albicans* was tested according to the brushed specimens (n = 2). Sterilized samples were submitted to the previously assigned brushing protocol, after which 15 mg of each toothpaste slurry or of distilled water was applied to the respectively treated surface. Afterwards, the samples were placed individually in a 24-well plate containing a *C. albicans* (10 CFU/mL in 1 mL of RPMI 1640 + MOPS) solution for 24 h, at 37 °C, under sterile conditions. After this time, the liquid cultures were removed from the wells, and 1 mL of RPMI 1640 + MOPS was once more added to the samples to induce *C. albicans* growth until adherence on the resin surface, after which the cultures were stirred at 37 °C for 24 h. After this process was completed, the surface of the resin containing the *C. albicans* culture was seeded using a sterile swab, in Petri dishes containing the Sabouraud dextrose agar (SDA) culture medium. The dishes were incubated at 37 °C for 24 h to ensure growth of the fungus and its potential CFUs^29^. This analysis was performed in duplicate.

### Inhibition of *Candida albicans* using the agar-well diffusion method

As described above, the surface of the Petri dishes filled with SDA was inoculated with an inoculum of 1 × 10^6^ CFU of *C. albicans* under the agar surface. A circular well (diameter of 6 mm) was punched in each plate with a sterile, stainless steel cork borer. A 100-µL aliquot of each toothpaste slurry or distilled water was added to the well. The plates were incubated under aerobic conditions, and kept in an upright position at 37 °C for 24 h. The zone of inhibition (ZOI) was measured after incubation^30–32^. This analysis was also performed in duplicate.

### Statistical analysis

The results were submitted to an exploratory analysis, the roughness, gloss and variation of roughness data were assessed using the generalized linear model, once they have presented normal distribution. The variation of gloss data did not show normal distribution and were submitted to the Kruskal Wallis and Dunn tests, followed by the correction with the Holm–Bonferroni method. The level of significance was set at 5%. All the analyses were performed using R software (R Core Team (2021); R Foundation for Statistical Computing, Vienna, Austria).

## Results

**Figure 1:**
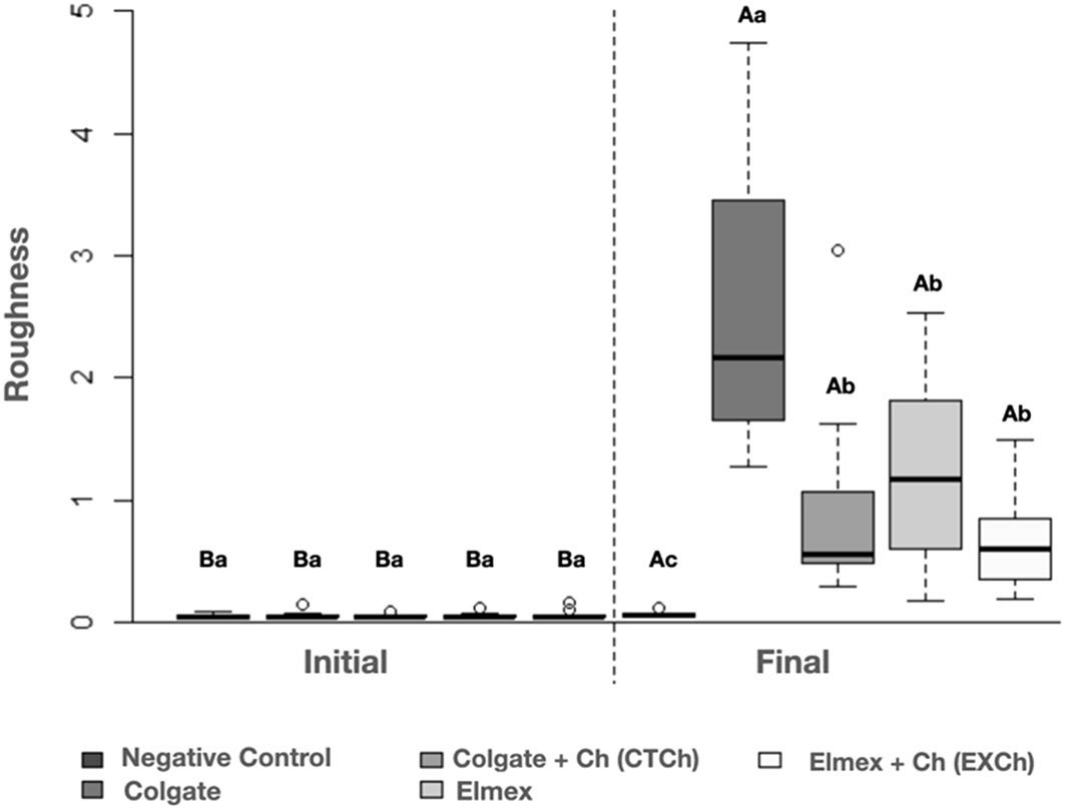
Analysis of roughness (µ) considering the evaluation before (initial) and after (final) application of the brushing protocols. The groups are presented in the following sequence in the graph: Negative control, Colgate, Colgate + Ch (CTCh), Elmex, Elmex + Ch (EXCh). (Uppercase letters indicate differences between the time points, while lowercase letters indicate differences among the groups.)

The roughness analysis results are available in Fig. 1 and Table 2. Figure 1 shows that there were no significant differences among the groups at the initial analysis, whereas all the groups were statistically different from the initial condition after brushing. Comparison of the groups at this time showed that all the groups treated with toothpaste had higher roughness values than the negative control (*p* = 0.002). Moreover, CT showed the highest roughness change, with significant differences in relation to the other groups (*p* < 0.0001). The addition of chitosan to CT (CTCh) resulted in a reduction in roughness without significant differences in relation to EX toothpaste (*p* < 0.05). Analysis of the variation in roughness (final vs. initial) observed in Table 2 shows the same differences among the groups.

**Table 2:** Comparison of roughness variations (µ) considering the different brushing protocols used (different letters indicate significant differences).

Group	Mean (standard deviation)	Median (min; max)
Negative control	0.01 (0.02)	0.02 (− 0.02; 0.04)^c^
CT	2.43 (1.11)	2.12 (1.24; 4.71)^a^
CTCh	0.90 (0.84)	0.50 (0.20; 3.01)^b^
EX	1.14 (0.78)	1.12 (0.14; 2.50)^b^
EXCh	0.63 (0.42)	0.56 (0.16; 1.34)^b^
*p* value	*p* < 0.0001	

**Figure 2:**
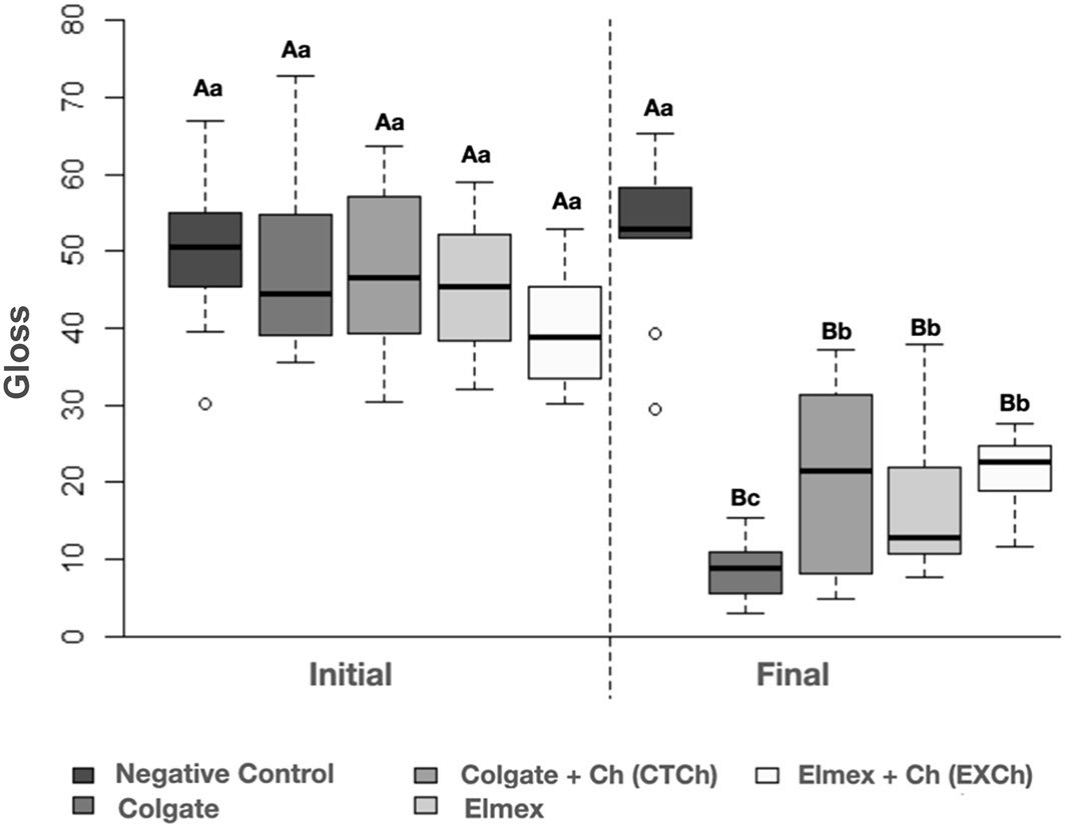
Analysis of gloss (GU) considering the evaluation before (initial) and after (final) application of the brushing protocols. The groups are presented in the following sequence in the graph: Negative control, Colgate, Colgate + Ch (CTCh), Elmex, Elmex + Ch (EXCh). (Uppercase letters indicate differences between the time points, while lowercase letters indicate differences among the groups.)

Results of the gloss surface analysis are shown in Fig. 2 and Table 3. There were no differences among the groups in the initial analysis, whereas all the groups were statistically different from the initial condition (*p* < 0.0001) after brushing, except NC. At this time point, all the toothpastes had a significantly lower degree of gloss in relation to NC (*p* < 0.05). CT had the highest gloss surface change, with statistically significant differences in relation to the other toothpastes (*p* < 0.05). The addition of chitosan to CT (CTCh) resulted in the same gloss changes observed for EX and EXCh (*p* > 0.05). Analysis of the variation in gloss (final vs. initial) in Table 3 shows that the differences among the groups is the same.

**Table 3:** Comparison of the gloss variations (GU) considering the different brushing protocols used (different letters indicate significant differences).

Group	Mean (standard deviation)	Median (min.; max.)
Negative control	2.08 (7.42)	2.63 (− 15.33; 11.60)^a^
CT	− 39.04 (10.76)	− 35.31 (− 58.80; − 27.43)^c^
CTCh	− 26.17 (17.53)	− 24.24 (− 52.78; − 0.05)^bc^
EX	− 27.77 (10.13)	− 28.76 (− 45.40; − 11.30)^bc^
EXCh	− 19.09 (8.88)	− 19.58 (− 32.20; − 6.00)^ab^
*p* value	*p* < 0.0001	

**Figure 3:**
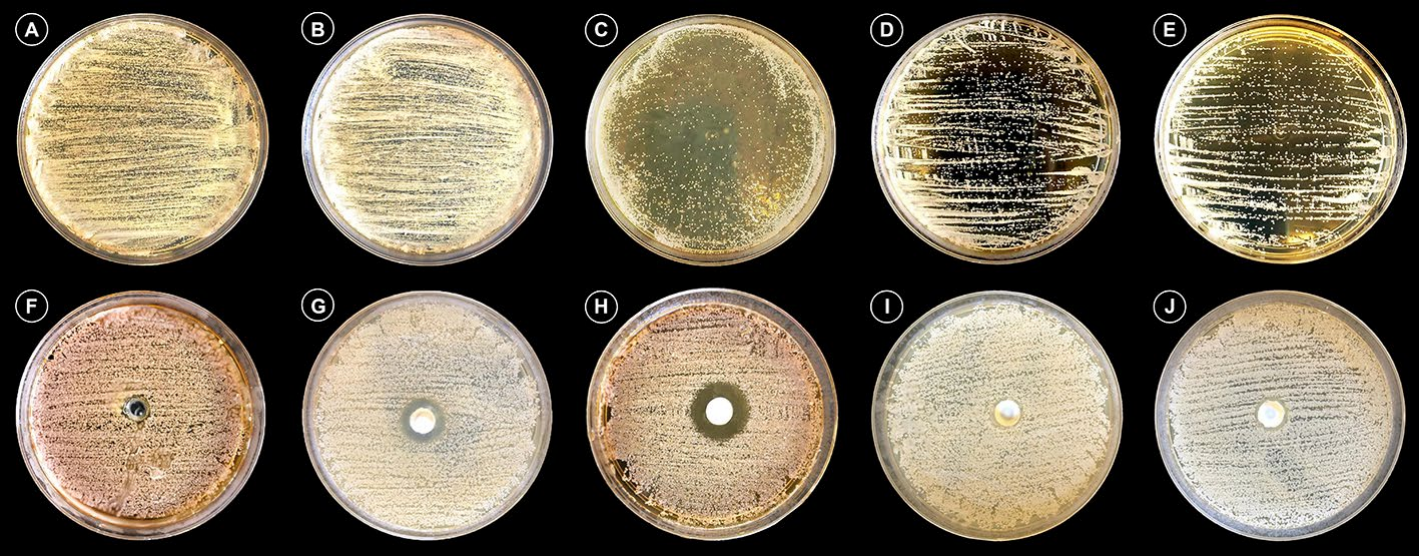
Antifungal analysis considering the adherence method (**a**–**e**) and the agar-well diffusion method, as well as the zone of inhibition (**f**–**h**): (**a**, **f**) negative control (distilled water); (**b**, **g**) CT; (**c**, **h**) CTCh; (**d**, **i**) EX; (**e**, **h**) EXCh.

The antifungal analysis was tested qualitatively (Fig. 3). As for the incidence of *C. albicans* on the surface of the brushed specimens, all the groups showed substantial growth in the microorganism, with CFUs higher than 300 UFC/ml. The incidence in decreasing order was: negative control > CT > EX = EXCh > CTCh. Analysis of the *C. albicans* inhibition potential shows that the addition of chitosan to CT was effective in increasing antimicrobial activity, given that the ZOI for CT was clearly higher than that of the other groups. The ZOI in decreasing order was: CTCh > CT > EX = EXCh = NC.

## Discussion

This study evaluated the effect of different toothpastes with or without chitosan, after long-term simulated toothbrushing. All the potential factors involved in the abrasive mechanism of the acrylic resin, such as number, rigidity, shape and tufts of the bristles, toothbrush load, and number of strokes, were standardized for all the groups. The only varying factor was the toothpaste used. A negative control brushed with distilled water was included for comparison purposes. The null hypotheses were rejected, considering that some differences were found in the brushing-related roughness and gloss analyses. The longevity of oral health in patients who wear a dental prosthesis most commonly involves chemical and/or mechanical methods of denture cleansing. The mechanical protocol of hygiene uses toothbrushing to remove biofilm on the prosthesis surface, mostly with soap or toothpaste^21^. In the case of fixed prostheses, toothpastes are recommended. Laboratory conditions are simulated to test brushing protocols, using simulating machines that standardize the position and load, and control the number of strokes, considering that a mean of 10,000 strokes is equivalent to 1 year of prosthesis surface cleaning, and that patients brush their dentures twice a day^21,33,34^.

Toothbrushing with distilled water (NC) caused a minor change in roughness and gloss in relation to the initial analysis, unlike the other groups, which all varied significantly in relation to the baseline. No differences were found between the initial versus final measurements for NC, hence implying that the NC had significantly higher gloss and lower roughness in relation to the other groups. This result shows that the interaction of the toothpaste ingredients with the acrylic surface at the base of the prosthesis is required to change the characteristics of the roughness and gloss. These data are important to bear in mind, considering that the action of the toothpastes on the roughness and gloss of the acrylic resin changed depending on the addition or non-addition of chitosan. These results corroborate those of a study that considered brushing with coconut soap as adequate for cleaning and maintaining a polished surface at the base of dentures in the long term^35,36^. Moreover, some other studies attest to the stability of this soap in contact with chemical solutions^37,38^, considering that thermopolymerizable resins show a higher monomeric conversion degree, and better physical properties, like roughness, gloss, color stability, and others^39,40^.

On the other hand, the inclusion of toothpastes in the brushing protocol impacts the physical properties (roughness and surface gloss) of the brushed acrylic resin, as previously described^17,20,34,41^. Actually, the combined use of toothpastes and brushes is the most popular and widespread method of practicing oral hygiene^3^, especially when the denture cannot be removed (implanted support protocols). Moreover, this is a normal habit practiced by most of the population, and has bearing on a person’s quality of life. Toothpastes have a formula rich in different ingredients, and their harsh effects on the surface of dental materials such as acrylic resin is mainly explained by their abrasive compounds^36^. Toothpastes are formulated with a different variety and number of ingredients, and different degrees of quality and abrasiveness. The most commonly used parameter to compare toothpaste abrasiveness is the Relative Dentin Abrasivity (RDA) method, which associates the abrasive potential of the toothpastes to dentin. It is common to discuss the effect of toothpaste as being harsh to dental materials, but there is no specific index to measure this harshness. Both silica-based toothpastes researched in this study had RDA values that could be considered safe (less than 250, according to the American Dental Association), inasmuch as CT had higher abrasiveness than EX^42^. In the present investigation, brushing with all the toothpastes tested resulted in negative changes in roughness and gloss, compared with the negative control (brushing with distilled water). Despite the action of the toothpaste slurries used (renewed repeatedly during the brushing protocol), the fact that brushes can assumedly retain toothpaste within their filaments implies that they add to the abrasiveness of the process^43^. Hence, the role of the specific toothpastes in altering the results becomes evident.

Considering the brushing protocols used, it can be seen that all the toothpastes increased the roughness to a level higher than 0.2 μm, a result related to the increased adhesion of microorganisms^44^. Accordingly, a change in the gloss to a level greater than 20 GU can be considered as high^45^, and all the toothpastes showed this variation except EXCh, which showed a mean of 19.09 GU (± 8.88). The CT group showed the highest change in roughness (with substantial significance in relation to the other toothpastes) and gloss (significantly different from EXCh alone). However, considering the final results for both analyses, the positive impact of chitosan on this toothpaste must be highlighted. In addition to containing the same abrasive agent as EX, CT also has a larger variety of ingredients, and probably a higher solid content. This would explain its higher RDA value, and its influence on the brushing effects^46^. Interestingly, chitosan showed higher potential in preventing roughness and gloss alteration in the toothpaste with the greater number of ingredients. This attests to its power of interaction with different active compounds, and its result regarding film-forming and lubricating ability^47,48^. As for the NC, although no significant variation was observed in roughness (higher than 0.2 μm), the gloss variation in the brushed resin was higher than 2 GU, a value considered clinically detectable by the human eye^45^.

To the best of the authors’ knowledge, there are no reports that test CT and EX toothpastes with or without chitosan for brushing acrylic-based surfaces. Although both toothpastes are silica-based^42^, classified CT as a very strong abrasive (> 80), and EX as just a strong abrasive (60–80). The results for the CT group attest to its abrasive properties against the acrylic material, given that it exhibited the highest alteration of roughness and gloss compared with the other products tested, especially EX toothpaste (significant difference). According to the study cited above, the results of using CT to brush different denture prosthesis materials, including thermopolymerizable resin, indicated a change in roughness similar to that of the present results, evidenced by groves on the surface of materials after treatment^17,20^. The addition of chitosan microparticles to this toothpaste impacted roughness and gloss, causing their reduction, similar to the results for EX. The addition of chitosan to EX produced no beneficial effect. In view of these data, it can be assumed that chitosan reduced the abrasive effect of CT toothpaste.

Chitosan is a molecule with positive zeta potential, and can electrostatically bind to negatively charged molecules^49^, such as the abrasives contained in toothpastes^47,48^. This interaction can favor the deposition and stabilization of the chitosan nanolayers on the surface, reduce the impact of certain forces^47,49^, particularly abrasive, and produce a lubricating effect^23^. Chitosan is a deacetylates derivative of chitin, the second most available biopolymer in nature, abundant in crustacean shells^26^. In addition to these properties, chitosan is a biocompatible and nontoxic molecule, whose potential can be explored in many fields of industry^26,50^. Another property of chitosan worthy of mention is its antimicrobial activity of importance to the fields of pharmaceuticals and medicine^26^. Chitosan has shown antifungal action against the adherence of *C. albicans* to the surface of acrylic resin^24,27^, as corroborated in the present results.

Regarding the potential of *C. albicans* adhesion under surfaces treated with different brushing protocols, this study showed that the growth of this fungus was substantially higher under the acrylic resin brushed with CT, compared to the other toothpastes. Although CT contains antifungal compounds like as triclosan^51^, the analysis performed by this study took into account the proliferation of microorganisms under the brushed surface. As expected, the presence of the *C. albicans* was more significant on the surface that had the greatest change in roughness. This is in agreement with the reports in the literature stating that irregularities under the surface of prosthesis bases can lead to *C. albicans* accumulation^1,3,13^*.* In the case of CT, the high roughness found in the present results is probably related to its abrasive potential (RDA 100 ± 5), and to its higher solid content, as discussed earlier. In the case of EX toothpaste, its RDA value was lower (60 ± 5), and *C. albicans* adhesion was clearly lower. Just as the addition of chitosan microparticles reduced the abrasiveness of CT, it also reduced the adhesion of *C. albicans*, thus corroborating the possible correlation between roughness and the adhesion potential of microorganisms, and attesting the antifungal effect of chitosan.

The agar-well diffusion method was performed to test the antifungal potential of the tested toothpastes individually. ZOI was higher for CT than EX toothpaste. Even though CT is more abrasive, a comparison of the two products shows that they share some antimicrobial-related compounds, such as sodium hydroxide, lauryl sodium sulfate and triclosan^38,52^. Triclosan has a well-established antifungal potential when used in mouth rinses^46,51^, but had no abrasive force in the present study, because it was used as a slurry. The incorporation of chitosan into CT increased the ZOI, thus highlighting its antifungal potential, and its possible synergistic effect in combination with other antimicrobials, like triclosan. Future investigations should be conducted to better explore the effects of this association.

Limitations from this study include the fact that the samples are not treated with salive to evaluate the formation of a *C. albicans* biofilm under a circumstance more similar to an in vivo conditions. However, despite of this, the antifungal potential of the toothpaste used were evidenced, indicating that are still points to be investigated in the future, considering the association with human saliva. Another limitation is the presentation of a qualitative result for the antifungal analysis. For sure, a larger sample would be more insights around the formation of *C. albicans*. Neverthelees, the present results performed after pilot tests are relevant to consider the potential of brushing with different toothpastes agains the formation of *C. albicans* biofilm.

In the present study, chitosan was used with a medium molecular weight, and at a concentration of 0.5%. This formulation of chitosan has been previously incorporated into toothpaste without altering its composition or properties, while promoting other related beneficial effects, such as antibacterial and remineralizing properties^25,53–55^. Importantly, in this study, chitosan was used in the form of a microparticle, in order to provide a hydrosoluble molecule, which makes it easier to be mixed with the toothpaste, without having to mix it with another vehicle, like acetic acid. The results showed the efficacy of associating chitosan with CT toothpaste. Previous investigations have been proposed to develop chitosan-based products for use in preventing and treating stomatitis resulting from the use of denture adhesive^24^. However, this use of chitosan would be impractical in the case of patients wearing fixed acrylic-based prostheses. In this respect, the present study presents a low-cost, easy-access procedure with a high potential for cleaning and disinfecting fixed or removable partial or total dentures.

## Conclusion

The type of toothpaste used for cleaning an acrylic prosthesis surface influences its roughness, gloss and fungal retention. The present results found that the abrasiveness of toothpaste is crucial to changing the degree of roughness and gloss, while clearly potentiating the presence of *C. albicans* on the surface. The inclusion of the chitosan microparticle in a toothpaste with higher abrasiveness and a higher number of ingredients or higher solid content, including triclosan, positively impacted the hygiene protocol tested, reducing the harsh effect of the toothpaste on roughness and gloss, and promoting the inhibition of *C. albicans*.

## Data Availability

The datasets used and analysed during the current study are available on reasonable request from Núbia Pini—nubiapini01@gmail.com.
